# The effect of pulse steroid treatment of ten days’ long on the improvement of bacillary layer detachment in a patient with Vogt-Koyanagi Harada disease

**DOI:** 10.22336/rjo.2021.36

**Published:** 2021

**Authors:** Ferdane Atas, Mahmut Kaya, Ali Osman Saatci

**Affiliations:** * Department of Ophthalmology, Dokuz Eylul University, Izmir, Turkey

**Keywords:** bacillary layer detachment, exudative retinal detachment, optical coherence tomography, uveitis, Vogt-Koyanagi-Harada disease

## Abstract

**Purpose:** To illustrate the improvement pattern of bacillary layer detachment (BLD) in a closely monitored patient with Vogt-Koyanagi-Harada (VKH) disease.

**Methods:** Imaging with color fundus photography and spectral domain optical coherence tomography (SD-OCT).

**Results:** The pattern of BLD was noticed better with each passing day under the treatment of ten days’ long pulse methylprednisolone (1 g/ day) therapy. Though a meaningful decrease in size and shape of the BLD occurred on the eight day of pulse treatment, it showed resolution at two weeks follow-up, but the associated subretinal serous fluid persisted until the sixth week of treatment.

**Conclusion:** The term BLD has become a widely used description as an OCT finding in some diseases but its evolution with the treatment was less illustrated previously. Thereby, our aim was to share our observation of a patient with VKH disease having BLD, with the ophthalmic community.

## Introduction

Historically, the term bacillary layer was coined for the inner and outer segments of the retinal photoreceptor layer as a histological description [**1**]. Nowadays, the term bacillary layer detachment (BLD) is also used in optical coherence tomography (OCT) nomenclature but the exact topographic correspondence of the retinal splitting remains somewhat controversial among the clinicians [**2**-**6**]. Recently, Ciccinelli et al. [**5**] argued that the splitting might occur at the level of inner segment myoid or within the ellipsoid zone or at the level of inner segment/ outer segment junction [**5**]. The presence of BLD has been reported in various clinical conditions such as toxoplasma retinochoroiditis, Vogt-Koyanagi-Harada (VKH) disease, peripapillary pachychoroid disease, acute posterior multifocal placoid pigment epitheliopathy and trauma with the help of OCT [**2**-**6**]. 

## Case Report

**Fig. 1:**
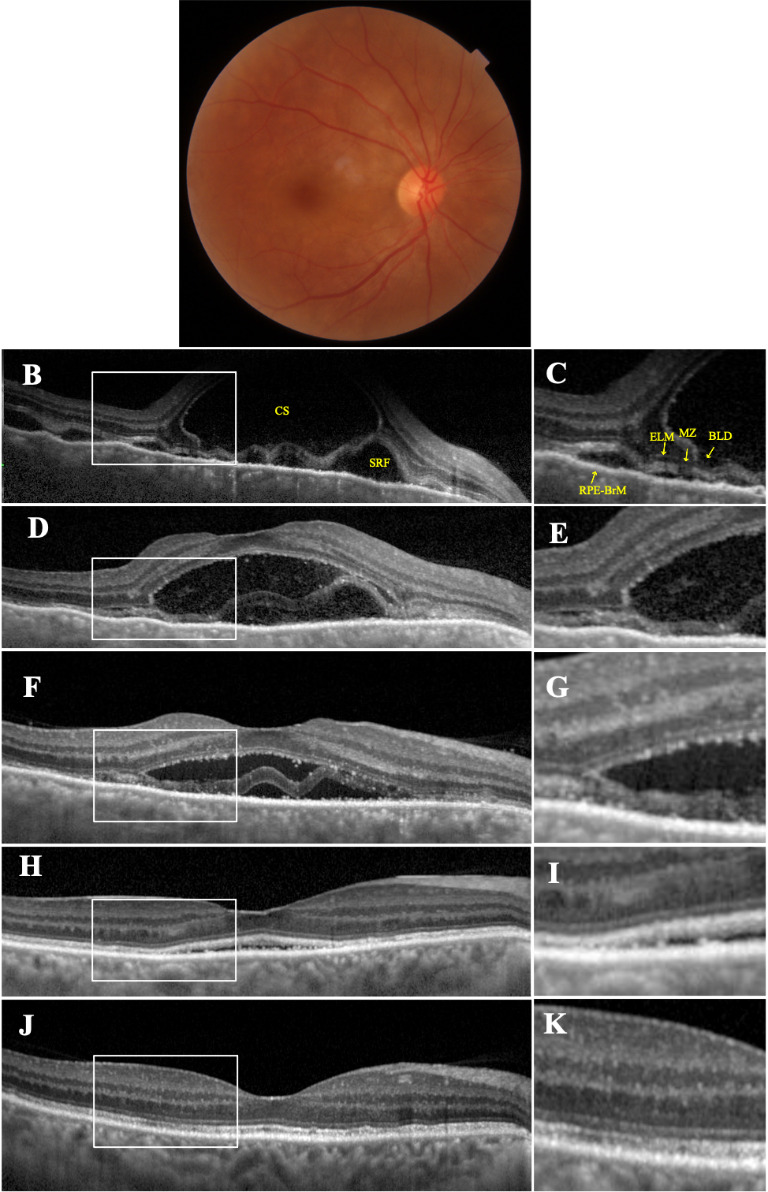
Right eye At presentation. Color fundus picture **(A)** showing the extent of serous retinal detachment at the posterior pole. Spectral-domain optical coherence tomography (SD-OCT) section **(B)**, delineating the massive subretinal fluid (SRF) and a large septum with a cavitary space (CS). The magnified image **(C)** shows the location of splitting. Outer retinal detachment is at the level of the myoid zone (MZ). The hyporeflective line corresponding to external limiting membrane (ELM) is anterior to the CS. The ellipsoid zone (EZ) is irregular and the remnants of photoreceptor segments lie anterior to the CS. The outer segment (OS) and interdigitation zone (IZ) remain adherent to the retinal pigment epithelium - Bruch’s membrane (RPE-BrM). Fourth day of pulse steroid therapy (PST). OCT section **(D)** and the its magnified view **(E)** demonstrating the decrease of SRF height and CS size with a more pronounced appearance of the splitting of the outer retinal layer borders. Eight day of PST. SD-OCT **(F)** and its magnified view **(G)** showing the emerging hyperreflective material around the bacillary layer detachment (yellow arrows) Two weeks later, SD-OCT **(H)** and its magnified view **(I)** showing the resolution of BLD with a small amount of residual SRF. Six weeks later - almost normalized foveal contour **(J)** and its magnified image **(K)** highlighting the intact looking outer retinal layer

**Fig. 2:**
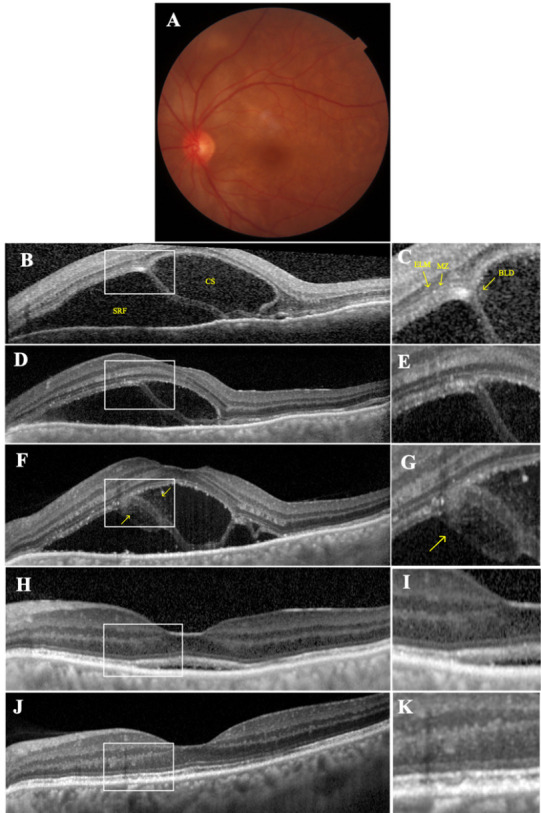
Left eye At presentation. Color fundus picture **(A)** showing the extent of serous retinal detachment at the posterior pole. Spectral-domain optical coherence tomography (SD-OCT) section **(B)**, delineating the massive subretinal fluid (SRF) and a large septum with a cavitary space (CS). The magnified image **(C)** shows the location of splitting. Outer retinal detachment is at the level of the myoid zone (MZ). The hyporeflective line corresponding to external limiting membrane (ELM) is anterior to the CS. The ellipsoid zone (EZ) is irregular and the remnants of photoreceptor segments lie anterior to the CS. The outer segment (OS) and interdigitation zone (IZ) remain adherent to the retinal pigment epithelium - Bruch’s membrane (RPE-BrM). Fourth day of pulse steroid therapy (PST). OCT section **(D)** and the its magnified view **(E)** demonstrating the decrease of SRF height and CS size with a more pronounced appearance of the splitting of the outer retinal layer borders. Eight day of PST. SD-OCT **(F)** and its magnified view **(G)** showing the emerging hyperreflective material around the bacillary layer detachment (yellow arrows) Two weeks later, SD-OCT **(H)** and its magnified view **(I)** showing the resolution of BLD with a small amount of residual SRF. Six weeks later - almost normalized foveal contour **(J)** and its magnified image **(K)** highlighting the intact looking outer retinal layer

A previously otherwise healthy 50-year-old woman presented with a sudden onset of bilateral visual deterioration and severe headache of two days’ duration on the New Year’s Eve. At presentation, her visual acuity was 20/ 125 OU. Slit-lamp examination yielded a few anterior chamber cells without any keratic precipitates and with trace cells in the vitreous, bilaterally. Fundus examination revealed bilateral optic disc hyperemia and exudative retinal detachment at the posterior pole (**Fig. 1A** and **Fig. 2A**). Spectral domain optical coherence tomography (SD-OCT; Spectralis®, Heidelberg Engineering, Heidelberg 2, Germany) disclosed a very high multilobular serous retinal detachment with septa in association with a pachychoroid appearance OU (**Fig. 1B,C** and **Fig. 2B,C**). Systemic examination and laboratory workup were carried out as fast as possible to rule out any systemic association, but with no positive finding. A diagnosis of incomplete VKH disease was established according to the revised diagnostic criteria set for the diagnosis of VKH disease [**7**]. OCT-monitored intravenous pulse methylprednisolone (1 g/ day) was administered for 10 days and this treatment was followed by oral prednisolone (64 mg/ day) with adalimumab (40 mg biweekly after the loading dose of 80 mg).

OCT scans began to reveal a splitting of the inner retina at the level of myoid zone (MZ) in accordance with the reduction in serous retinal detachment height on the fourth day of the pulse steroid treatment in both eyes (**Fig. 1D,E** and **Fig. 2D,E**). The intact external limiting membrane (ELM) and anterior retinal structures were displaced anteriorly. Retina pigment epithelium (RPE) - Bruch membrane complex was observed as a continuous hyperreflective band at the base of the BLD. BLD improved gradually on the eight day of the pulse steroid treatment with a hyperreflective material around the BLD (**Fig. 1F,G** and **Fig. 2F,G**). Though the BLD showed resolution within two weeks (**Fig. 1H,I** and **Fig. 2H,I**), residual subretinal fluid did not vanish until the sixth week of the treatment. But fortunately, inner, and outer segments of the photoreceptors almost fully recovered (**Fig. 1J,K** and **Fig. 2J,K**).

## Discussion

The BLD is an OCT finding that can be observed in the acute phase of VKH disease. As briefly mentioned above, the exact site of splitting is still under debate, and, in the present case, the splitting of photoreceptor layer took place at the level of the inner myoid zone, just underneath the external limiting membrane. In a recent paper by Aggarwal et al. [**6**], BLD was noted in 112 eyes of 118 eyes (94.9%) with acute VKH disease in association with the serous retinal detachment at presentation. The external limiting membrane that was present at the anterior aspect of the BLD showed focal discontinuity in eight of the 112 eyes (7.1%) with BLD. The interdigitation zone at the base of the BLD showed discontinuity in 53 of 112 eyes (47.3%) with BLD. In 102 of 112 eyes (91.1%), the ellipsoid zone could not be identified as a separate hyperreflective line at the base of the BLD. Bacillary layer detachment showed resolution within 3.4 ± 1.3 days after the initiation of intravenous methylprednisolone therapy. 

## Conclusion

The implication of BLD in the anatomic and functional outcome and the impact of its improvement speed are still unknown in patients with VKH disease. To our best knowledge, the effect of pulse steroid treatment on the improvement pattern of BLD was not illustrated in detail previously and thereby we wanted to share our observation with the ophthalmic community. 

**Conflict of Interest**

The authors state no conflict of interest. 

**Informed Consent and Human and Animal Rights statement**

An informed consent was obtained from the patient included in this Case Report. 

**Authorization for the use of human subjects**

Ethical approval: The research related to human use complies with all the relevant national regulations, institutional policies, is in accordance with the tenets of the Helsinki Declaration, and has been approved by the Ethics Committee of Dokuz Eylul University, Izmir, Turkey.

**Acknowledgements**

None. 

**Sources of Funding**

None. 

**Disclosures**

None.
